# The Money Buffer Effect in China: A Higher Income Cannot Make You Much Happier but Might Allow You to Worry Less

**DOI:** 10.3389/fpsyg.2016.00234

**Published:** 2016-02-23

**Authors:** Bin Li, Aimei Li, Xiaotian Wang, Yunsong Hou

**Affiliations:** ^1^The Institute of Enterprise Development, Jinan UniversityGuangzhou, China; ^2^Management School, Jinan UniversityGuangzhou, China

**Keywords:** money buffer effect, subjective well-being, emotional well-being, income, material affluence, social class, positive affect, negative affect

## Abstract

This study examined the possibility that there is a curvilinear relationship between income and subjective well-being in China. This study also investigated whether this curvilinear relationship is moderated by social class and mediated by respondents' material affluence. The study was conducted in China, and the sample consisted of 900 blue-collar workers and 546 white-collar workers. The results for emotional well-being showed that income significantly predicted negative affect but not positive affect. This finding indicates that in China, high incomes may not make people happier but might allow them to worry less, which we call the “money buffer effect.” The results also showed that material affluence mediates the interaction effect between income and social class on subjective well-being. The implications of these results for future research and practice are discussed.

## Introduction

Could higher income make you much happier? It hasn't a definitely answer for this question yet. Many studies have found that income has a weak positive or no relationship with subjective well-being (e.g., Easterlin, 1974; Diener and Biswas-Diener, 2002; Kahneman et al., 2006; Ahuvia, 2008; Caporale et al., 2009; Park, 2009; Sing, 2009; Yao et al., 2009). However, these studies have some limitations.

First, most research that explores the relationship between income and subjective well-being relies on evaluations of life. Recently, researchers have tried to separate subjective well-being into cognitive and emotional components, and the results showed that people with above-average income were higher evaluation of their lives but were barely higher emotional well-being (Kahneman and Deaton, 2010). An evaluation of life could represent only the cognitive side of subjective well-being, or emotional well-being could be divided into two measures: positive affect and negative affect. Thus, income might have different impacts on cognitive and emotional well-being, or even on positive and negative affect. Unfortunately, little has been known about relationship between income and differential emotional well-being. The present study sought to shed some light in this respect.

Second, several theories suggest that higher income cannot bring higher subjective well-being due factors such as social comparisons (Sweeney and McFarlin, 2004; Mentzakis and Moro, 2009) and income aspirations (Solberg et al., 2002; Bjørnskov et al., 2008; Brown et al., 2009; McBride, 2010). All of these explanations may reflect one key factor—individual subjective well-being is dependent on how one subjectively experiences material affluence. Thus, this study aims to more directly measure individuals' subjective material affluence to determine whether it plays a key role in the relationship between income and subjective well-being.

Third, previous studies have found that social class differences in subjective well-being, the means of subjective well-being were lower for people from lower middle class (Lachman and Weaver, 1998; Bedin and Sarriera, 2015). It is possible that part of what is advantage of upper class is that their higher income. But in terms of theories of social comparison and income desire, upper class is flowed by the higher reference income and higher desire, which is meaning that the actual income would make differential experiences of material affluence and subjective well-being between differential social class. Addressing this, the impact of social class on income and subjective well-being was proposed and explicitly examined in this study.

## Theory and hypotheses

### Income and subjective well-being

Subjective well-being is most commonly measured by asking people for a global evaluation of their life (Kahneman et al., 2006). A growing body of empirical evidences have shown that income has a weak positive relationship with subjective well-being (Diener and Biswas-Diener, 2002; Kahneman et al., 2006; Caporale et al., 2009; Diener, 2009). In fact, some studies have demonstrated that income had no relationship with subjective well-being (Ahuvia, 2008; Park, 2009; Sing, 2009; Yao et al., 2009). Recent research shows that subjective well-being has both cognitive and affective components (emotional well-being; Kahneman and Deaton, 2010). The cognitive component refers to the individual's overall life evaluation, and the affective component refers to the presence of positive emotions and the absence of negative emotions (Tay et al., 2015). Kahneman and Deaton (2010) found that income was positively and closely related to life evaluation but not to emotional well-being, emotional well-being also rose with log income but did not rise beyond an annual income of $75,000.

Therefore, there maybe have an inverted U-shaped relationship between income and emotional well-being. However, emotional well-being, which includes positive affect and negative affect, is not measured by a single metric but by different metrics. Positive affect is not the opposite of negative affect (Cacioppo and Berntson, 1999; Cacioppo and Gardner, 1999), that is, when a person has a low level of sadness, this does not mean he has a high level of happiness. Moreover, someone can feel positive affect and negative affect at the same time. Thus, income may differentially affect individuals' positive and negative affect. This research will look deeper into the relationship between income and life evaluations, especially of emotional well-being. Therefore, we proposed the following hypothesis:

*Hypothesis 1. There is a differential relationship between income and life evaluation, positive affect, and negative affect: an inverted U-shaped relationship between income and evaluation of life, and positive affect, but a U-shaped relationship between income and negative affect*.

### Material affluence and subjective well-being

But why can't higher income make higher subjective well-being? Over the past 40 years, numerous studies have discussed the relationship between income and subjective well-being. Social comparison theory is one of the most fruitful and most important for applications to the income-happiness relationship. At the individual level, the most important thing is not absolute income but relative income. Especially at higher levels of income, relative income has a stronger effect on subjective well-being than absolute income (Sweeney and McFarlin, 2004; Mentzakis and Moro, 2009). When someone takes a higher income as the reference object, he is in the inferior position and will then feel less happiness (Ball and Chernova, 2008; Bjørnskov et al., 2008; Smyth et al., 2010). That is, in upward comparisons, individuals feel poorer, which reduces subjective well-being. However, in downward comparisons, individuals feel more affluent, which increases subjective well-being.

Consistent with processes of social comparison, individual income aspirations increase with their own incomes as well as with the average income in the community in which they live (Stutzer, 2004). Many previous studies have found that higher income aspirations reduce people's subjective well-being, which depends only on the gap between the aspirational income and actual income rather than on the income level as such (Solberg et al., 2002; Stutzer, 2004; Bjørnskov et al., 2008; Brown et al., 2009; McBride, 2010). When the aspirational income is higher than actual income, the subjective experience of material affluence is much lower, which leads to less subjective well-being. Therefore, material affluence may play a mediation role in the relationship between income and subjective well-being. Therefore, we hypothesize that:

*Hypothesis 2. Material affluence mediates the relationship between income and subjective well-being*.

### Social class and subjective well-being

Other recent research has found that economic growth is not associated with increase in happiness when accompanied by growing income inequality (Napier and Jost, 2008; Brockmann et al., 2009; Oishi and Kesebir, 2015). Income inequality leads to relative deprivation, which turns people into frustrated achievers—those who achieve higher incomes in absolute terms but are dissatisfied with their income positions relative to the winners (Brockmann et al., 2009). Income inequality occurs across social classes in China. Blue-collar workers (members of the lower class) have lower income, fewer social resources, and lower perceived social status, a combination that affords them less personal control and increases dependence on others to achieve their desired outcomes (Argyle, 1994; Domhoff, 1998). Conversely, white-collar workers (members of the upper class) are characterized by economic independence, elevated personal control, and freedom with respect to subjects of personal choice (Snibbe and Markus, 2005; Stephens et al., 2007). However, some researchers suggest that the relation between income and subjective well-being depends on the amount of material desires that people's income allows them to fulfill (Diener and Biswas-Diener, 2002). The idea that income enhances subjective well-being only insofar as it helps people meet their basic needs (Diener and Biswas-Diener, 2002), which is meaning that higher income has a stronger impacts on subjective well-being in lower class than upper class.

However, the theory of desire posits that the desire for money increases quicker than income rises; thus upper class may have bigger gap between desire and actual income than lower class (Solberg et al., 2002; Stutzer, 2004; Bjørnskov et al., 2008; Brown et al., 2009; McBride, 2010), then upper class may possibly feel less subjective well-being than lower class. Thus, we hypothesize that:

*Hypothesis 3. Social class moderates the relationship between income and material affluence, and subjective well-being*.

## Methods

### Participants

Employees in the same industrial zone in Guangzhou City in the Province of Guangdong, China were categorized as either white-collar workers or blue-collar workers. After we eliminated invalid responses, 900 blue-collar workers and 546 white-collar workers remained in our sample. The sample consisted of 51.5% blue-collar male workers and 45.4% white-collar male workers ranging in age from 16 to 43 years (*M* = 24.00, *SD* = 5.42). Written informed consent was obtained from all participants before starting the investigation in accordance with the Declaration of Helsinki, and the study was approved by the ethical committee of Jinan University.

### Measures

#### Income

Household annual income was divided into six levels: level 1 for under 50,000 RMB (45.3% of the total sample), level 2 for 50,000–80,000 RMB (28.6%), level 3 for 80,000–120,000 RMB (16.8%), level 4 for 120,000–200,000 RMB (6.1%), level 5 for 200,000–300,000 RMB (2.3%), and level 6 for over 300,000 RMB (0.9%).

#### Material affluence

This measure used eight items derived from the Material and Time Affluence Scale (Kasser and Sheldon, 2009), which employs items rated on a 7-point scale from 1 (strongly disagree) to 7 (strongly agree), such as “I have been able to buy what I want.” Cronbach's alpha in this study was 0.81.

#### Subjective well-being

A discussion of subjective well-being must recognize the distinction between the concepts of a life evaluation and emotional well-being (Kahneman and Deaton, 2010). The evaluations of life were conducted using Cantril's Self-anchoring Striving Scale, which has the respondent rate his or her current life on a ladder on which 0 is “the worst possible life for you” and 10 is “the best possible life for you” (Kahneman and Riis, 2005). Emotional well-being was considered in two parts to assess both positive affect and negative affect. Respondents were asked questions about the presence of various emotions during the previous day; positive affect included enjoyment, happiness, and pleasure, and negative affect included sadness, stress and worry (Kahneman and Deaton, 2010). All items were rated on a 6-point scale from 1 (strongly disagree) to 6 (strongly agree). In this study, Cronbach's alpha was 0.88 for positive affect and 0.87 for negative affect.

## Results

### Description and correlation analysis

The means, standard deviations, and Pearson correlations of the variables are presented in Table 1. We found that income was significantly related to evaluation of life (*r* = 0.50, *p* < 0.01), positive affect (*r* = 0.07, *p* < 0.05), emotional well-being (*r* = 0.22, *p* < 0.01), and material affluence (*r* = 0.07, *p* < 0.05) and was significantly negatively related to negative affect (*r* = −0.21, *p* < 0.01). Material affluence was significantly positively related to evaluation of life (*r* = 0.09, *p* < 0.01), positive affect (*r* = 0.11, *p* < 0.01), and emotional well-being (*r* = 0.12, *p* < 0.01) and significantly negatively related to negative affect (*r* = −0.08, *p* < 0.01).

**Table 1:** **Means, standard deviations, and bivariate correlations between variables**.

**Variable**	**1**	**2**	**3**	**4**	**5**	**6**	**7**	**8**	**9**	**10**
1 Sex										
2 Age	−0.091^**^									
3 Education	−0.208^**^	0.050								
4 Income	−0.050	−0.273^**^	0.165^**^							
5 Social Class	0.057^*^	−0.029^**^	0.058^*^	0.363^**^						
6 Material affluence	−0.040	−0.088^**^	−0.008	0.285^**^	0.098^**^					
7 Evaluation of life	−0.027	−0.112^**^	0.165^**^	0.501^**^	0.180^**^	0.088^**^				
8 Emotional well-being	0.011	−0.123^**^	0.026	0.182^**^	0.132^**^	0.124^**^	0.215^**^			
9 Positive affect	0.050	−0.213^**^	−0.016	0.066^*^	0.219^**^	0.111^**^	0.116^**^	0.773^**^		
10 Negative affect	0.037	−0.042	−0.054^*^	−0.209^**^	0.028	−0.078^**^	−0.210^**^	−0.747^**^	−0.156^**^	
*M*	1.51	24.00	2.51	0.93	1.38	20.30	5.11	0.92	3.88	2.95
*SD*	0.50	5.42	0.97	1.09	0.49	5.43	1.70	1.71	1.15	1.10

* *p < 0.05*,

** *p < 0.01*.

### Relationship between income and subjective well-being

Table 2 presents the results of the hierarchical regression analysis used to test our hypotheses. After centering our independent variables (Aiken and West, 1991), we introduced the control variables into the regression equation (step 1) and then introduced the main effect variable, income, into the equation (step 2). Next, to test our prediction that income has a curvilinear relationship with evaluation of life, we introduced quadratic income in step 3. As shown in Table 2, the coefficient associated with this term is statistically significant (β = −0.15, *p* < 0.001, Δ*R*^2^ = 0.01, *p* < 0.001): An inverted U-shaped relationship between income and evaluation of life was observed.

**Table 2:** **Curve regressions of evaluation of life on income**.

**Variable**	**Step1**	**Step2**	**Step3**
Sex	0.01	0.02	0.03
Age	0.11^***^	0.05	0.04
Education	0.12^***^	0.07^**^	0.05^*^
Social Class	0.18^***^	0.00	0.02
Income		0.50^***^	0.58^***^
Income^2^			−0.15^***^
Δ*R*^2^	0.07	0.20	0.01
Δ*F*	25.10^***^	360.72^***^	19.26^***^

* *p < 0.05*,

** *p < 0.01*,

*** *p < 0.001*.

**Figure 1:**
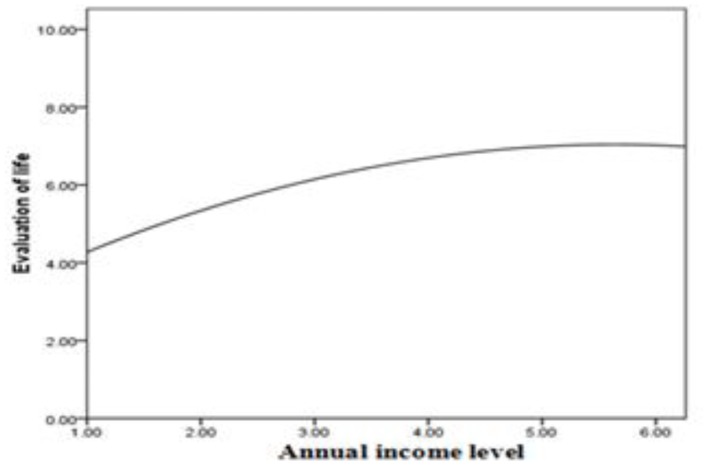
**Curvilinear effect of income on evaluation of life**.

Figure 1 shows that the relationship between income and evaluation of life is described by an inverted U-shaped function for employees. Employees with higher annual incomes reported better evaluation of life.

**Figure 2:**
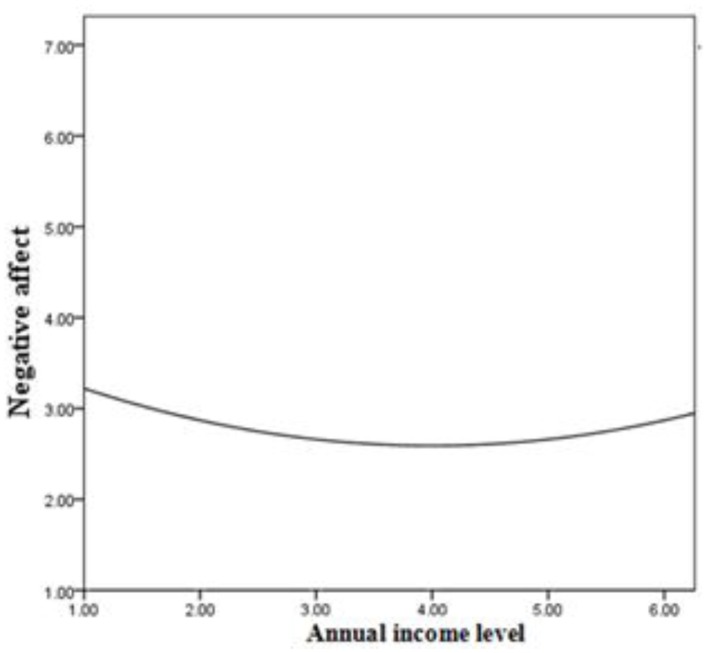
**Curvilinear effect of income on negative affect**.

We used the same approach to test our prediction that income has curvilinear relationships with both positive affect and negative affect. As shown in Table 3 and Figure 2, a U-shaped relationship between income and negative affect was observed (β = 0.12, *p* < 0.001). However, the relationship between income and positive affect did not follow an inverted U-shaped path (β = −0.001, *n.s*.), nor was it linear (β = 0.001, *n.s*.).

**Table 3:** **Curve regressions of negative affect on income**.

**Variable**	**Step1**	**Step2**	**Step3**
Sex	0.02	0.01	0.01
Age	−0.02	0.01	0.02
Education	−0.04	−0.02	0.00
Social Class	0.03	0.11^***^	0.10^***^
Income		−0.25^***^	−0.33^***^
Income^2^			0.12^**^
Δ*R*^2^	–	0.05	0.01
Δ*F*	1.36	71.27^***^	9.55^**^

** *p < 0.01*,

*** *p < 0.001*.

### Mediation analysis

We hypothesized that material affluence would mediate the inverted U-shaped relationship between income and life evaluation. To test this hypothesis, we used 3 regression models, as shown in Table 4 (Baron and Kenny, 1986). M1 showed that the coefficient associated with income and material affluence was statistically significant (β = 0.14, *p* < 0.001). M2 showed that both the relationship between income and life evaluation (β = 0.58, *p* < 0.001) and the relationship between income^2^ and life evaluation (β = −0.15, *p* < 0.001) were statistically significant. M3 showed that when material affluence was introduced, it was significantly related to life evaluation (β = 0.14, *p* < 0.001); although both the relationship between income and life evaluation (β = 0.54, *p* < 0.001) and the relationship between income^2^ and life evaluation (β = −0.14, *p* < 0.001) remained significant, their significance was greatly reduced. Thus, material affluence partially mediated the relationship between income and life evaluation.

**Table 4:** **Curve regressions of evaluation of life on income: Mediation in material affluence**.

**Variable**	**M1: Material affluence**	**M2: Evaluation of life**	**M3: Evaluation of life**
Sex	−0.06^*^	0.03	0.03
Age	0.02	0.04	0.05^*^
Education	0.02	0.05^*^	0.07^**^
Social Class	0.02	0.02	0.03
Income	0.14^***^	0.58^***^	0.54^***^
Income^2^	−0.09	−0.15^***^	−0.14^***^
Material affluence			0.14^***^
Δ*R*^2^			0.02
Δ*F*			33.76^***^

* *p < 0.05*,

** *p < 0.01*,

*** *p < 0.001*.

We also tested the hypothesis that material affluence would mediate the U-shaped relationship between income and negative affect, as shown in Table 5. M3 showed that when material affluence was introduced, it was significantly related to negative affect (β = −0.07, *p* < 0.001). Both the income-negative affect relationship (β = −0.30, *p* < 0.001) and the income^2^-negative affect relationship (β = −0.12, *p* < 0.001) remained significant but greatly reduced. Thus, material affluence partially mediated the relationship between income and negative affect.

**Table 5:** **Curve regressions of negative affect on income: Mediation in material affluence**.

**Variable**	**M4: Material affluence**	**M5: Negative affect**	**M6: Negative affect**
Sex	−0.06^*^	0.01	0.01
Age	0.02	0.02	0.01
Education	0.02	0.00	−0.01
Social Class	0.02	0.10^***^	0.10^**^
Income	0.14^***^	−0.33^***^	−0.30^***^
Income^2^	−0.09	0.12^**^	0.12^**^
Material affluence			−0.07^**^
Δ*R*^2^			0.01
Δ*F*			6.72^*^

* *p < 0.05*,

** *p < 0.01*,

*** *p < 0.001*.

Because researchers have suggested that both the method developed in Baron and Kenny (1986) and the Sobel test (Sobel, 1982) suffer from low statistical power in most situations (Preacher and Hayes, 2004), we used a bootstrapping method, which is considered a more powerful approach to estimating indirect effects in simple mediation models (Preacher and Hayes, 2004). In this study, we repeated the bootstrapping process using the recommended minimum 5000 repetitions. The results of the multiple regression and bootstrapped analysis are presented in Table 6. The total effect of income on life evaluation was significant (0.47), the direct effect of income on life evaluation was significant (0.39), and most importantly, the mediating effect of material affluence on income and evaluation of life was significant (0.08), with a 95% bootstrapped confidence interval (CI) of 0.04–0.12. Moreover, there were significant total effects (−0.16), direct effects (−0.11), and indirect effects through material affluence (−0.05) on the relationship between income and negative affect [95% CI = −0.08 ~ −0.02]. Income did not have a significant total effect or a direct effect on positive affect but may have an indirect effect through material affluence (0.08, 95% CI = 0.04 ~ 0.11). A significant indirect effect on emotional well-being (0.13, 95% CI = 0.08 ~ 0.19) was found when using bootstrapping to detect mediation effects.

**Table 6:** **Regressions of well-being on income: Meditation in material affluence**.

**Regression model**	**Unstandardized regression coefficients**		**Bootstrap procedure**	
	**Total effect**	**Direct effect**	**Indirect effect**	**BC95%CI**
Income-MA-EL	0.47^***^	0.39^***^	0.08^***^	0.04 ~ 0.12
Income-MA-EWB	0.21^***^	0.08	0.13^***^	0.08 ~ 0.19
Income-MA-PA	0.04	−0.04	0.08^***^	0.04 ~ 0.11
Income-MA-NA	−0.16^***^	−0.11^***^	−0.05^***^	−0.08 ~−0.02

*MA, Material affluence; EL, Evaluation of life; EWB, Emotional well-being; PA, Positive affect; NA, Negative affect*.

*** *p < 0.001*.

### Moderation analysis

We hypothesized that social class would moderate the inverted U-shaped relationship between income and life evaluation. To test this hypothesis, as shown in Table 7, we controlled for linear interactions (income × social class) in step 4 and introduced the relevant quadratic-linear interaction (income^2^ × social class) in step 5 of the regression equation. The coefficient associated with this interaction term was statistically significant (β = −0.62, *p* < 0.001).

**Table 7:** **Curve regressions of evaluation of life on income: Moderation in social class**.

**Variable**	**Step1**	**Step2**	**Step3**	**Step4**	**Step5**
Sex	0.02	0.02	0.03	0.03	0.04
Age	0.11^***^	0.05	0.04	0.05^*^	−0.01
Education	0.13^***^	0.07^**^	0.05^*^	0.07^**^	−0.01
Social class		–	0.02	0.03	0.11^***^
Income		0.49^***^	0.58^***^	0.65^***^	0.73^***^
Income^2^			−0.15^***^	−0.01	0.49^***^
Social class × Income				−0.23^***^	−0.25^***^
Social class × Income^2^					−0.62^***^
Δ*R*^2^	0.04	0.23	0.01	0.02	0.02
Δ*F*	17.67^***^	209.25^***^	19.26^***^	38.63^***^	35.20^***^

* *p < 0.05*,

** *p < 0.01*,

*** *p < 0.001*.

**Figure 3:**
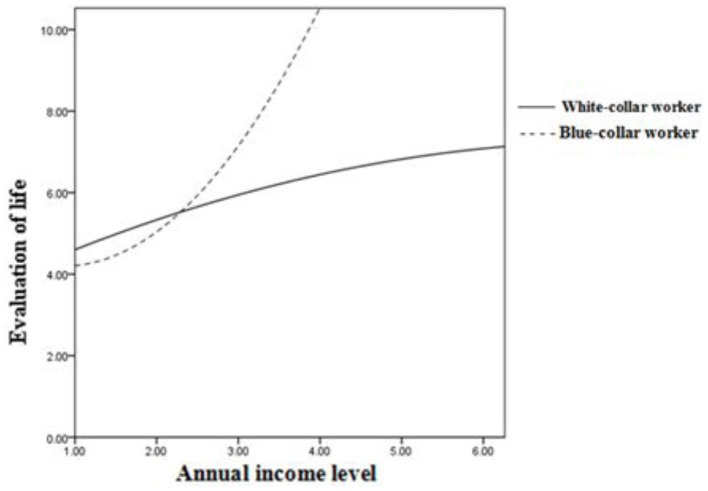
**Income and evaluation of life: Moderation by social class**.

With respect to white-collar workers, Figure 3 shows that there was an inverted U-shaped relationship between income and evaluation of life (β = −0.10, *p* < 0.05); however, there was a U-shaped relationship between income and evaluation of life for blue-collar workers (β = 0.17, *p* < 0.001).

We also hypothesized that social class would moderate the U-shaped relationship between income and negative affect. To test this hypothesis, Table 8 shows that we controlled for the linear interaction (income × social class) in step 4 and introduced the relevant quadratic-linear interaction (income^2^ × social class) in step 5. The coefficient associated with the linear interaction term was statistically significant (β = 0.12, *p* < 0.05).

**Table 8:** **Curve regressions of negative affect on income: Moderation of social class**.

**Variable**	**Step1**	**Step2**	**Step3**	**Step4**	**Step5**
Sex	0.02	0.01	0.01	–	–
Age	−0.02	0.01	0.02	0.03	0.04
Education	−0.04	−0.02	–	0.02	0.02
Social class		0.12^***^	0.10^***^	0.120^***^	0.09^*^
Income		−0.25^***^	−0.32^***^	−0.36^***^	−0.38^***^
Income^2^			0.12^***^	0.05	−0.09
Social class × Income				0.12^**^	0.12^***^
Social class × Income^2^					0.17
Δ*R*^2^	0.03	0.05	0.01	0.01	–
Δ*F*	1.48^***^	36.185^***^	9.53^**^	7.44^**^	1.90

* *p < 0.05*,

** *p < 0.01*,

*** *p < 0.001*.

**Figure 4:**
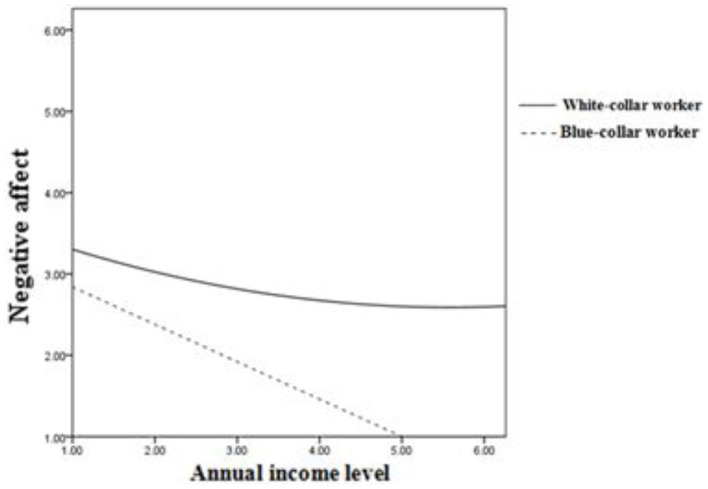
**Income and negative affect: Moderation by social class**.

With respect to white-collar workers, Figure 4 shows that there was a U-shaped relationship between income and negative affect (β = 0.02, *p* < 0.05) and that there was no U-shaped relationship (β = −0.04, *n.s*.). However, a negative linear relationship (β = −0.29, *p* < 0.05) between income and negative affect was observed for blue-collar workers.

### Mediated moderation models

Finally, we predicted that material affluence would mediate the interaction between income and social class on evaluation of life. As shown in M9 of Table 9, when material affluence was introduced, it was significantly related to evaluation of life (β = 0.12, *p* < 0.001). The linear (income × social class; β = −0.23, *p* < 0.001) and the quadratic-linear (income^2^ × social class; β = −0.57, *p* < 0.001) interactions remained significant but were greatly reduced. Thus, material affluence partially mediated the interaction effect between income and social class on evaluation of life.

**Table 9:** **Regressions of evaluation of life on income: Mediated moderation model**.

**Variable**	**M7: Material affluence**	**M8: Evaluation of life**	**M9: Evaluation of life**
Sex	−0.06^*^	0.04	0.03
Age	0.02	−0.01	–
Education	0.02	−0.01	0.01
Social class	0.02	0.11^***^	0.11^***^
Income	0.14^***^	0.73^***^	0.70^***^
Income^2^	−0.09	0.49^***^	0.45^***^
Social class × Income	0.26^***^	−0.25^***^	−0.23^***^
Social class × Income^2^	0.05	−0.62^***^	−0.57^***^
Material affluence			0.12^***^
*R*^2^	0.09	0.30	0.32
Δ*R*^2^			0.12^***^
Δ*F*			24.16^***^

* *p < 0.05*,

*** *p < 0.001*.

M3 in Table 10 shows that when material affluence was introduced, it was significantly related to negative affect (β = −0.06, *p* < 0.001); the linear interaction (income × social class) with negative affect changed from a positive (β = 0.12, *p* < 0.001) to a negative (β = −0.11, *p* < 0.001) relationship. Thus, material affluence partially mediated the interaction effect between income and social class on evaluation of life.

**Table 10:** **Regressions of negative affect on income: Mediated moderation model**.

**Variable**	**M10: Material affluence**	**M11: Negative affect**	**M12: Negative affect**
Sex	−0.06^*^	–	0.05
Age	0.02	0.04	0.03
Education	0.02	0.02	0.02
Social class	0.02	0.09^*^	0.09^*^
Income	0.14^***^	−0.38^***^	−0.35^***^
Income^2^	−0.09	−0.09	−0.07
Social class × Income	0.26^***^	0.12^***^	−0.11^**^
Social class × Income^2^	0.05	0.17	0.14
Material affluence			−0.06^*^
*R*^2^	0.09	0.06	0.07
Δ*R*^2^			0.01
Δ*F*			5.15^*^

* *p < 0.05*,

** *p < 0.01*,

*** *p < 0.001*.

## Discussion

This study intended to explore whether income significantly affects subjective well-being between social classes. We found that income has no significant effect on positive affect but that income does have significant effects on negative affect and evaluation of life. These findings suggest that higher incomes will not create positive affect but will reduce negative affect. Furthermore, the results show that material affluence plays a mediating role in the relationship between income and subjective well-being and that social class has a moderating effect on the relationship between income and subjective well-being. Based on these results, a high income might have a cushioning effect: Money does not increase positive affect, but it can reduce negative affect.

There are three theoretical contributions of this research. First, our research contributes to the body of research on the income-happiness relationship in China by considering the differential effects on positive emotional well-being versus negative emotional well-being rather than the difference between cognitive well-being and emotional well-being. To date, scholars have shown that income has a weak positive relationship with subjective well-being (Easterlin, 1974; Diener and Biswas-Diener, 2002; Kahneman et al., 2006; Caporale et al., 2009). Studies have found that the different ways of measuring happiness may lead to these findings; these studies have also found that income has significant effects on cognitive well-being but not on emotional well-being (Kahneman and Deaton, 2010). However, recent studies have found that positive affect and negative affect are two different types that function independently. In other words, someone can experience positive affect and negative affect simultaneously. For example, when someone receives an inheritance following the death of a partner or family member, she might be simultaneously sad for the personal loss and happy for the financial gain. Therefore, we argued that these two emotions, which are components of well-being, may be differentially affected by income. A higher income may not increase workers' positive affect but might provide a buffer against negative affect. The results support the hypothesis that income has no significant effect on positive affect but does have significant effects on both negative affect and evaluations of life. A U-shaped relationship between income and negative affect was observed. By contrast, an inverted U-shaped relationship between income and evaluation of life (cognitive well-being) was observed. Why are people in China who have higher incomes not necessarily happier than those with lower incomes? Some research has noted that how people spend money is a key factor in determining whether money increases subjective well-being. Perhaps money does increase subjective well-being because people do not spend their money correctly (Dunn et al., 2011). For example, some people frequently spend money on material items rather than on experiences or spend money on themselves instead of buying for others. Spending on big-ticket or luxury items does not produce long-term happiness; instead, spending on small items brings pleasure (Aaker et al., 2011). Therefore, there are many rich people who are not happy. In addition, Chinese people would rather save money than spend money, a cultural trait that is evidenced by China's highest personal savings rate in the world. From 1978 to 2014, Chinese savings deposits increased from 21.06 billion yuan to 44.17 trillion yuan. The average annual growth in savings during that time was 59%, far outpacing GDP growth. By contrast, the average global savings rate is only 19.7%. The Chinese savings rate was 46% in 2005 and 51% in 2007; in contrast, the U.S. savings rate was 0.5% in 2005 and under 2% in 2007. Since the 2008 financial crisis, the U.S. personal savings rate has risen to 4.5%, whereas China's has remained at 38.3% since 2008. The Chinese love to save money and do not like to spend money. Therefore, money cannot increase rich people's happiness in China.

However, money can provide a buffer against negative affect, not least because having money allows people to stop worrying about survival and material matters. When the pressure for basic goods decreases, negative affect is also reduced. In recent years, some researchers (Kesebir and Hong, 2008; Zhou et al., 2009) have suggested that money provides a buffer against pain. Zhou and Gao (2008) argue that social support is the first buffer against mental pain and that money provides the next buffer. In other words, when people fail to secure social support, they turn to money to ease their psychological pain. Baumeister et al. (2008) have noted that money can provide a buffer against psychological pain because money itself can provide social support. Money acts as a social resource in its pain relief role. Social resources provide support—particularly in threatening situations. Thus, money can improve an individual's overall response capacity, reducing the need for other social resources. In other words, money can help ward off negative affect to some extent. Therefore, a money buffer effect might exist: higher incomes cannot make people much happier but can allow them to worry less. This paper contributes to this perspective by showing that separating the negative affect and positive affect components of emotional well-being is important and necessary, thereby enhancing our understanding of the relationship between income and subjective well-being.

Second, this research provides a fresh perspective on the relationship between income and subjective well-being in China by examining material affluence as a mediator. A number of previous studies have examined how income influences subjective well-being (Solberg et al., 2002; Stutzer, 2004; Sweeney and McFarlin, 2004; Bjørnskov et al., 2008; Brown et al., 2009; Mentzakis and Moro, 2009; McBride, 2010). Those studies, however, have focused on how income directly affects well-being. Previous studies have found that psychological factors play important roles in the relationship between income and happiness. The issue is not how much money people earn but how rich they feel. Therefore, this research focused on material affluence as the mediating factor between income and subjective well-being. Across studies, the results partially support the hypothesis that material affluence acts as a mediator between income and subjective well-being. Material affluence partially mediated the inverted U-shaped relationship between income and evaluation of life and partially mediated the U-shaped relationship between income and negative affect. Although income was not shown to significantly influence positive affect, material affluence had a direct effect on positive affect. Rich people are few in number; most people are ordinary but live their lives with happiness. Regardless of how much money they have, if individuals feel that they have enough, they are happy. This finding indicates that in terms of relative income, the feeling of material affluence may be a key factor in happiness, which indicates an opportunity to integrate the theories of desire and of social comparison.

The theory of desire posits that happiness depends on the income gap between desired and real income, not just the actual level of income. If people desire more money as their income rises, they will never have what they want; their real happiness will always be lower than they expect it should be (Solberg et al., 2002; Stutzer, 2004; Bjørnskov et al., 2008; Brown et al., 2009; McBride, 2010). This theory further posits that the desire for money increases more quickly than income rises; thus, if people feel that they are not materially affluent, they will not obtain happiness from their money. Social comparison theory holds that if individuals compare themselves to people with lower incomes than theirs, they will be happy. However, they will be unhappy if they compare their themselves to those with higher incomes than theirs (Sweeney and McFarlin, 2004; Mentzakis and Moro, 2009). This position emphasizes that comparing oneself to people who are poorer leads to feelings of increasing material affluence, which produces happiness. However, when a person compares himself to people who are richer than he is, his feeling of material affluence is low, and he feels unhappy. Therefore, money can buy happiness depending on how rich people feel rather than on how much money they have. A subjective feeling of richness is much more important than the actual amount money possessed (provided that it is enough money to meet basic needs). Therefore, material affluence is an important factor affecting the relationship between income and subjective well-being. This research constitutes the first examination of this linkage, combining the theories of desire and comparison regarding material affluence, a mediator of the relationship between income and subjective well-being. This is a unique contribution to the literature. The findings are also consistent with the traditional view that to Chinese people: “Happiness is contentment.”

A further contribution of our research concerns the social class effects of income for white-collar workers and blue-collar workers on well-being in China. Social class is a multifaceted construct that includes income, education, and occupation (Kraus and Stephens, 2012). In China, social class is even more complex, involving not only education but also insurance pensions and other sources of income. In this study, social class examined by dividing respondents into blue-collar workers and white-collar workers. Our results showed that social class moderated the relationship between income and subjective well-being. In the case of white-collar workers, there was an inverted U-shaped relationship between income and life evaluation and a U-shaped relationship between income and negative affect. This finding is inconsistent with the previous literature (Kahneman and Deaton, 2010). However, for blue-collar workers, there was a U-shaped relationship between income and life evaluation, and there was a negative linear relationship, rather than a U-shaped relationship, between income and negative affect. Blue-collar workers in China have fewer social resources and lower social status. Indeed, they must rely on acquiring more money to make their lives more secure; thus, when a blue-collar worker's income increases, their life evaluation improves and negative affect decreases.

From a practical perspective, the results of this study indicate that high incomes might not create more positive affect but can reduce negative affect. More importantly, the current findings provide insight into the benefits of material affluence, which enables higher life evaluations and positive affect and reduces negative affect. These findings suggest that in addition to improving employees' annual incomes, ethical businesses might institute policies to improve the material affluence of their employees. Blue-collar workers need higher income at first. However, white-collar workers might require emotional rewards rather than higher income to improve their material affluence.

There are three limitations that should be noted. First, all data were collected using self-reporting questionnaires that were collected at a single point in time. Future research might use a longitudinal design to avoid common method bias and use experience sampling to assess the associations among income, material affluence, and subjective well-being. Second, although this sample was relatively large, it was likely not a representative sample of white-collar and blue-collar workers in China. Future research should expand the sample and include other countries with collectivist cultures, such as Japan. In addition, countries with individualist cultures, such as the U.S., should be evaluated to replicate the findings reported here in Western societies. Finally, our research was concerned with the effect of material affluence on the relationship between income and subjective well-being. How material affluence influences subjective well-being is not yet known. People with high material affluence also have more time affluence, higher quality social interactions, more power, more self-control, and more opportunities to attain flow experiences, which all improve subjective well-being. Future studies could also consider the psychological mechanisms behind material affluence.

## Author contributions

BL and AL conceived and designed experiments; BL and YH carried out experiments; BL and XW analyzed experimental results; BL and AL wrote the manuscript.

## Funding

This research was supported by grants from Humanity and Social Science Youth foundation of Ministry of Education of China (14YJCZH068), the National Natural Science Foundation of China (71571087, 71271101), the Fundamental Research Funds for the Central Universities(15JNLH005), National Natural Science Foundation of Guangdong in China (2014A030311022), and Postdoctoral Science Foundation of China(2015T80941, 2014M560696).

### Conflict of interest statement

The authors declare that the research was conducted in the absence of any commercial or financial relationships that could be construed as a potential conflict of interest. The reviewer, LP, and handling Editor declared their shared affiliation, and the handling Editor states that the process nevertheless met the standards of a fair and objective review.
